# Fluid management of cardiopulmonary bypass during pulmonary endarterectomy for CTEPH patients impacts perioperative outcome

**DOI:** 10.1016/j.jhlto.2025.100253

**Published:** 2025-06-04

**Authors:** K. Furrer, D. Bettex, T. Horisberger, I. Inci, N.G. Nagaraj, H.-T. Morselli, B. Battilana, R. Schuepbach, S. Ulrich, M. Hebeisen, I. Opitz

**Affiliations:** a Department of Thoracic Surgery, University Hospital Zurich, University of Zurich, Zurich, Switzerland; b Institute of Anesthesiology, University Hospital Zurich, Zurich, Switzerland; c Department of Perfusion, University Hospital Zurich, Zurich, Switzerland; d Institute of Intensive Care Medicine, University and University Hospital Zurich, Zurich, Switzerland; e Department of Pulmonology, University Hospital Zurich, Zurich, Switzerland; f Department of Biostatistics, Epidemiology, Biostatistics and Prevention Institute, University of Zurich, Zurich, Switzerland

**Keywords:** Pulmonary endarterectomy (PEA), Chronic thromboembolic pulmonary hypertension (CTEPH), Cardiopulmonary bypass (CPB), Crystalloid priming solutions, 5% human, Albumin (HA) priming

## Abstract

**Background:**

Pulmonary endarterectomy (PEA) using deep hypothermic circulatory arrest (DHCA) and cardiopulmonary bypass (CPB) is the preferred treatment for chronic thromboembolic pulmonary hypertension (CTEPH). Crystalloid priming solutions cause hemodilution with disadvantages, and no standard exists for PEA. This study evaluates CTEPH patient outcomes after adding 5% human albumin (HA) to the CPB-prime and hemodilution solution during PEA.

**Methods:**

The effect of the CPB-protocol change was evaluated by comparing patients operated under the old and new protocols in a 1:1 propensity score match based on age, sex, and preoperative peripheral vascular resistance (PVR).

**Results:**

Matching resulted in 56 patients (28 per group) between July 1, 2010, and May 31, 2020. The new protocol group had a lower intraoperative fluid balance (1.85 vs 6.76 liters, *p* < 0.001), vasoactive-inotropic score (VIS) (8.7 vs 17.7, *p* = 0.04), shorter operative- (407 vs 451 min, *p* = 0.03), and hospitalization time (LOS) (18 vs 27 days, *p* = 0.008). Morbidity and mortality at 30- and 90-days were similar in both groups. The new protocol was associated with reduced intraoperative fluid balance after adjustment for operative time (−3.7 liters [95% CI −5.2, −2.1], *p* < 0.0001). Lower intraoperative fluid balance was associated with shorter hospitalization in the intensive care unit, intubation time, LOS, and lower VIS (*p* = 0.0011, 0.0013, 0.008, <0.0001, respectively). The protocol change shortened LOS, independent of operative time, by 27% [95% CI, 44%, 6%], *p* = 0.02.

**Conclusions:**

Priming and maintenance protocol for CPB with the addition of 5% HA had a beneficial effect on intraoperative fluid balance and improved outcome after PEA for patients with CTEPH.

## Graphical abstract

**Figure:**
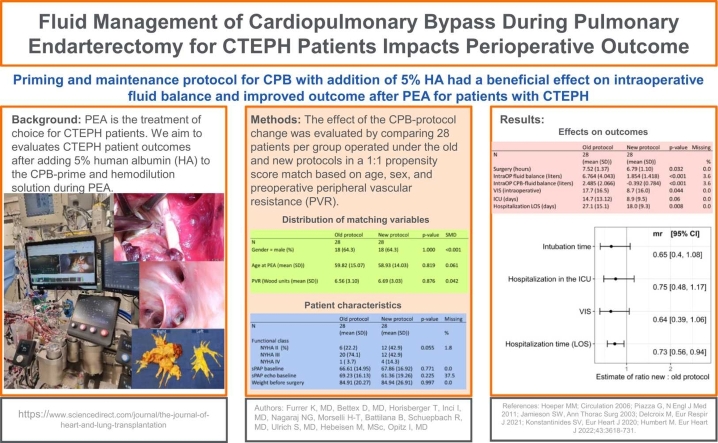

## Background

Chronic thromboembolic pulmonary hypertension (CTEPH) is a severe and often underdiagnosed disease associated with debilitating symptoms and high mortality.^1^ It is characterized by recurrent pulmonary thromboemboli leading to vascular scarring, remodeling, and progressive right heart failure.^2^ Pulmonary endarterectomy (PEA) is the treatment of choice and offers long-term relief of symptoms for patients with CTEPH.^3^, ^4^, ^5^, ^6^, ^7^, ^8^, ^9^ Over the past two decades, PEA using deep hypothermic circulatory arrest (DHCA) during cardiopulmonary bypass (CPB) has become the standard technique, yielding mortality rates below 5% with careful patient selection.^6^, ^10^, ^11^, ^12^, ^13^ Meticulous perioperative fluid balance is crucial to diminish capillary leak, pulmonary edema, and hypotension after PEA.^6^, ^13^ In PEA surgery, there is no generalized standard for CPB-prime solutions. The present study compares outcomes of patients with CTEPH after changing the prime and maintenance solutions protocol for CPB to balanced ringer acetate with 5% human albumin (HA), compared with balanced ringer acetate alone during elective PEA surgery.

## Materials and methods

This retrospective study, with 1:1 propensity score matching of two groups, is based on the registry of patients with CTEPH undergoing PEA at University Hospital Zurich (Switzerland). This project was approved by the Ethics Committee of University Hospital Zurich (Reference No.: 2020-02566 (RIO)) in accordance with the amended Declaration of Helsinki. All patients gave informed consent to use their personal data for research.

### Study participants

All patients with CTEPH undergoing PEA at University Hospital Zurich between July 1, 2010, and May 31, 2020, were considered for the analysis. Operability was decided by an interdisciplinary CTEPH team according to guidelines, assessing the presence of surgically accessible lesions and absence of high-risk comorbidities.^9^ PEA surgeries were performed under CPB with DHCA at 20 °C, with repetitive circulatory arrest limited to 20 min.^4^, ^14^

In early years, only few PEA procedures were performed, but from 2015 the center can be regarded as a medium-volume center performing between 9 and 15 PEA surgeries each year. While the PEA procedure remained consistent, the CPB-protocol for PEA surgery was changed in 2018 by adding 5% HA in prime and during CPB (1,000-1,500 ml 5%HA in total). The two analysis groups were formed according to this protocol change. CPB was established in a standard fashion between the ascending aorta and the superior and inferior vena cava. Body temperature was decreased to 20 °C before aortic cross-clamping, and hemodilution to a hematocrit ≤25% was reached before DHCA. Right and left endarterectomy were performed with circulatory arrests for intermittent reperfusion. Thromboembolic location was classified according to the Jamieson classification.^4^, ^15^

Perioperative management during CPB included routine hemofiltration, perioperative steroid prophylaxis, and the use of mannitol in the priming solution to maintain renal perfusion and prevent acute kidney injury (AKI). The CPB circuit and technical procedures, including the use of DHCA, were consistent across both protocol groups. Autologous priming was not systematically performed. Fluid balance was calculated as the net difference between fluids administered and removed during the perioperative period. It was measured at three stages: intraoperative fluid balance, postoperative fluid balance for days 1 and 2, and total fluid balance (sum of intraoperative and postoperative balances). Data were obtained from electronic medical records and verified by the perfusionist team.

Crystalloid volume administered intraoperatively was defined as the total volume of crystalloid solutions added to the CPB prime and administered during surgery. This measurement did not include postoperative crystalloid administration.

### Study design

Group A included participants who underwent PEA before the CPB-protocol change in September 2018 (previous CPB-protocol), whereas group B included patients operated under the new CPB-protocol. Propensity score matching was employed to pseudo-randomize participants and streamline retrospective data extraction from CPB-machine records. Participants from group B were 1:1 matched to their most similar counterparts from group A based on age, sex, and preoperative pulmonary vascular resistance (PVR). To address potential confounders due to the learning curve, operative time was included in all regression models, ensuring that the effect of the protocol change was independent of operative time.

### Preoperative characteristics

Comorbidities, CTEPH therapy, laboratory parameters, clinical and functional status, pulmonary function, and right heart catheterization data were collected.

### Perioperative characteristics

Duration of CPB, aortic cross clamping, circulatory arrest, intraoperative fluid balance, blood product substitution, operative time, intra- and post-operative inotropic (dobutamine) and catecholamine support (norepinephrine, epinephrine) (Table S2) as well as the necessity for extracorporeal membrane oxygenation (ECMO), were assessed. Administration of blood products followed local institutional practice in accordance with national recommendations regarding blood product use.^16^, ^17^, ^18^

### Outcomes

The primary outcome measure was duration of hospitalization in the intensive care unit (ICU, days), was chosen due to its clinical relevance in assessing recovery after PEA. ICU length of stay—hospitalization time (LOS) reflects the cumulative impact of perioperative factors, including fluid management, hemodynamic stability, and postoperative complications. Secondary outcomes included intraoperative fluid balance (liters), vasoactive-inotropic score (VIS) of catecholamines^19^ in the first 2 days after operation, intubation duration (days), hospitalization time (LOS, days), 30- and 90-day mortality and morbidity according to the Clavien-Dindo classification,^20^ systolic pulmonary arterial pressure (sPAP, mmHg), and New York Heart Association (NYHA) functional class after 6 months (above II or not).

### Statistical analysis

Descriptive statistics included means and standard deviations (SD) for normally distributed data, medians, and interquartile ranges (IQR) for skewed continuous variables, and numbers and percentages for categorical variables. Propensity score matching of surgeries with previous and new protocols was conducted using nearest neighbor matching of age, sex, and preoperative PVR values with the MatchIt package in R, Matched groups were compared using standardized mean difference (SMD).

Statistical tests comparing new and old protocols were applied to all peri- and post-operative patient characteristics, without adjustment for multiple testing. Continuous variables were compared using *t*-tests for means and Mann-Whitney *U* tests for medians, while categorical variables were compared using Fisher’s exact test.

Regression models were used to quantify the effect of the new CPB-protocol on long-term, short-term, and intermediate outcomes, adjusting for potential confounders such as operative time. The analysis also assessed whether fluid balance mediated the beneficial effect of the new protocol. Models adjusted for NYHA-class at baseline, while linear regression models used log-transformed outcomes for better fit, with resulting coefficients back-transformed to mean ratios (MR) for interpretation as percentage change. Logistic regression was used for binary outcomes (morbidity and NYHA-class), with odds ratios (OR) reported. It was not possible to analyze 30- and 90-day mortality due to few events, or sPAP due to substantial missing data. Regression coefficients were reported with 95% confidence intervals (CI). Models with outcomes: LOS, duration of hospitalization in the ICU, and intubation duration considered fluid balance as a mediator of the new protocol’s effect. The statistical analyses were performed using standard tools in R (version 4.0.3) and SPSS (version 26).^21^ Results were reported according to STROBE guidelines.^22^

## Results

**Figure 1:**
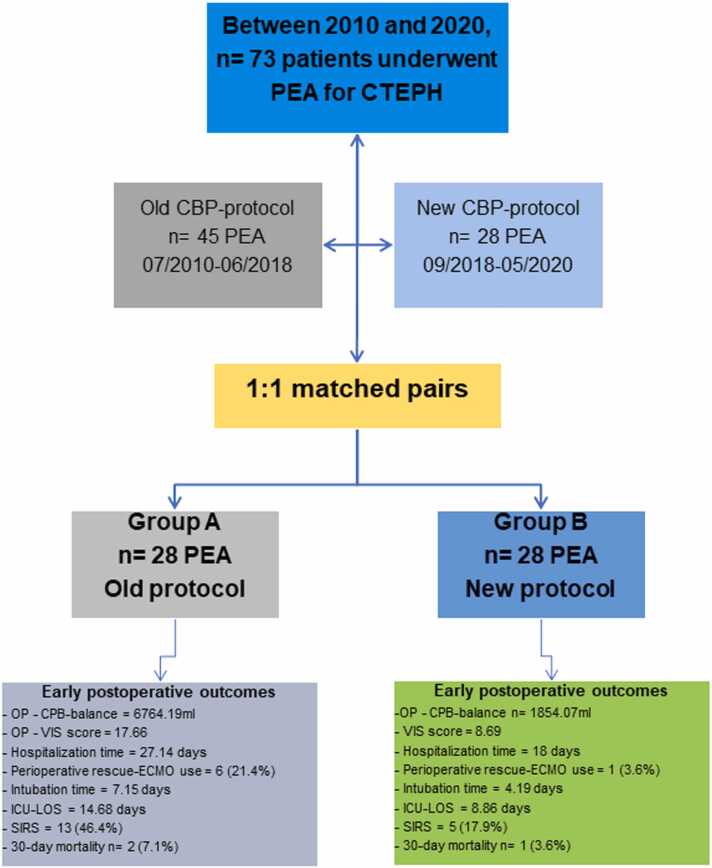
*Flow chart of the total cohort and the following two 1:1 matched cohorts and their respective early postoperative outcomes.* 28 patients operated with the new protocol were 1:1 matched with propensity scores to the 28 patients that were operated with the standard protocol. CTEPH, chronic thromboembolic pulmonary hypertension; CPB, cardiopulmonary bypass; ECMO, extracorporeal membrane oxygenation; ICU, intensive care unit, VIS, vasoactive-inotropic score, LOS, length of stay – hospitalization time.

Out of 73 eligible patients (Table S1), 56 were 1:1 propensity score matched, resulting in 28 patients per CPB-protocol group (Figure 1). The matching process effectively balanced the groups, with SMDs below 0.1 (Table 1). The analysis focused on comparing pre-, peri-, and postoperative characteristics between the two groups to evaluate the impact of the CPB-protocol change on patient outcomes. Most matched patients were operated in the years 2015 to 2020 (52 of 56) when the center was a medium-volume center.

**Table 1:** Distribution of Matching Variables in Matched Dataset According to Old and New Protocol

	Old protocol	New protocol	SMD*
	*n* = 28	*n* = 28	
Male sex, *n* (%)	18 (64.3)	18 (64.3)	<0.001
Age, years, mean (SD)	59.82 (15.07)	58.93 (14.03)	0.061
PVR**, WU, mean (SD)	6.56 (3.10)	6.69 (3.03)	0.042

Abbreviations: PVR, pulmonary vascular resistance; SMD, standardized mean difference; WU, wood units.

SMD values < 0.1 indicate that the matching was successful and the matched dataset is balanced with regard to the matching variables.

* SMD.

** PVR, expressed in WU = mmHg/L/min.

## Demographics and peri- and post-operative outcomes

Table 2 depicts preoperative patient characteristics in both protocol groups. The median age of patients was 60.5 years, IQR: [50.3, 72.0]. Table 3 compares the two protocol groups based on various peri- and postoperative patient characteristics.

**Table 2:** Comparative Preoperative Characteristics of Patients Operated According to Old and New Protocol

	Old protocol *n* = 28	New protocol *n* = 28
*Age, years*		
Median [QR]	61.5 [51.3, 71.8]	59 [49.3, 72.8]
Weight**kg		
Median [IQR]	83.5 [71.2, 88.7]	81.05 [65.4, 98]
*BMI, kg/m^2^*		
Median [IQR]	25.8 [24.1, 34.3]	25.5 [23.4, 29.5]
Mean (SD)	28.9 (7.5)	26.5 (4.9)
*History of deep venous thrombosis or acute pulmonary embolism, n (%)*	27 (96.4)	26 (92.9)
*Active or recent (<3 year) smoker, n (%)*	6 (21.4)	5 (17.9)
*NYHA functional class, n (%)*		
II	6 (22.2)	12 (42.9)
III	20 (74.1)	12 (42.9)
IV	1 (3.7)	4 (14.3)
*Thrombophilia or blood disorder, n (%)*	5 (17.9)	7 (25)
*ASA score, n (%)*		
III	12 (42.9)	2 (7.1)
IV	16 (57.1)	26 (92.9)
*Preoperative 6-min walk test distance, m*		
Median [IQR]	441 [290,507]	481 [420,554]
Mean (SD)	406 (129)	477 (111)
*Therapies*		
Treatment naïve, *n* (%)	12 (57.1)	17 (60.7)
Double therapy, *n* (%)	2 (7.1)	4 (14.3)
Prostanoid therapy, *n* (%)	0	1 (3.6)
Phosphodiesterase inhibitors, *n* (%)	1 (3.6)	0
Endothelin receptor antagonists, *n* (%)	8 (28.6)	7 (25)
Statins, *n* (%)	5 (17.9)	5 (17.9)
*Hemodynamics - preoperative RHA*		
*RAP, mmHg*	*n* = 28	*n* = 27
Median [IQR]	10.5 [7,14]	7 [5,10]
Mean (SD)	12.2 (8.6)	8.3 (5.6)
*sPAP, mmHg*		
Mean (SD)	66.32 (15.07)	67.92 (16.76)
*PAWP, mmHg*	*n* = 27	*n* = 28
Median [IQR]	13 [10,15]	10 [8,12]
Mean (SD)	12.22 (3.84)	10.32 (2.3)
*Cardiac index, L/min/m^2^*		
Median [IQR]	2.5 [2.2, 3]	2.3 [2.01, 2.77]
Mean (SD)	2.60 (0.61)	2.43 (0.55)
*Selected preoperative laboratory data*		
*Blood urea nitrogen, mmol/liter*	*n* = 27	*n* = 24
Median [IQR]	7.1 [5.3, 9.3]	5.5 [4.8, 7.4]
Mean (SD)	7.18 (2.35)	6.6 (2.85)
*Creatinine, µmol/liter*		
Median [IQR]	92 [81.5, 115.25]	91.5 [70.25, 99.25]
Mean (SD)	98.67 (25.20)	90.85 (23.04)
*Creatinine clearance, ml/min*		
Median [IQR]	64.5 [56.75, 81.75]	73.5 [61.25, 89.75]
Mean (SD)	69.64 (20.90)	74.40 (18.91)
*Pulmonary functional tests*		
*DLCO, %*	*n* = 27	*n* = 26
Median [IQR]	67.5 [56,80]	63 [58.25, 78.5]
Mean (SD)	69.52 (16.46)	65.78 (14.73)
*FEV1, %*		
Median [IQR]	79 [65.5, 100.75]	89 [66.25, 96.67]
Mean (SD)	81.85 (19.60)	82.77 (21.69)
*Resting O2, %*		
Median [IQR]	91.9 [89, 94.8]	92.1 [90,93]
Mean (SD)	90.55 (8.64)	91.15 (4.17)

Abbreviations: FEV1, Forced Expiratory Volume in 1 Second (lung airflow measure, DLCO, diffusing capacity for carbon monoxide); IQR, interquartile range; NYHA, New York Heart Association; PAWP, pulmonary arterial wedge pressure; PVR, pulmonary vascular resistance; RAP, right atrial pressure; RHA, right heart catheterization; SD, standard deviation; sPAP, systolic pulmonary arterial pressure.

Values are presented as mean (SD), median [IQR], or *n (%).*

** Before surgery.

**Table 3:** Comparative Peri- and Postoperative Characteristics of Patients Operated According to Old and New Protocol

	Old protocol *n* = 28	New protocol *n* = 28	*p*-value	Missing*
*Operative time, min*				
Mean (SD)	451 (82)	407 (66)	0.03	0.0
Associated cardiac procedure, n (%)**	1 (3.6)	2 (7.1)	1.000	0.0
*CPB duration, min*				
Median [IQR]	312 [280,330]	283[252,305]		
Mean (SD)	309(56)	282 (50)	0.02	0.0
*Aortic clamping duration, min*				
Median [IQR]	142 [114,162]	108 [92,133]		
Mean (SD)	139 (28)	113 (25)	<0.001	0.0
*Circulatory arrest duration, min*				
Median [IQR]	44 [38,58]	48[34,62]		
Mean (SD)	47 (13)	48 (15)	0.8	0.0
*Jamieson classification, n (%)*				
I right	6 (21.4)	9 (32.1)	0.45	0.0
II right	10 (35.7)	5 (17.9)		
III right	11 (39.3)	12 (42.9)		
IV right	1 (3.6)	2 (7.1)		
I left	2 (7.1)	3 (10.7)	0.14	0.0
II left	10 (35.7)	3 (10.7)		
III left	15 (53.6)	19 (67.9)		
IV left	1 (3.6)	3 (10.7)		
*Urine output without CPB (ml)*				1.8
Mean (SD)	707 (689)	436 (464)	0.092	
*Intraoperative fluid balance, ml*				
Mean (SD)	6764 (4044)	1854 (1418)	<0.001	3.6
*Intraoperative CPB-fluid balance, ml*				
Mean (SD)	2485 (2066)	−392 (784)	<0.001	3.6
*Cell saver balance, ml*				
Mean (SD)	1232 (839)	1002 (473)	0.215	3.6
*Crystalloid balance, ml*				
Mean (SD)	2964 (2444)	1603 (778)	0.007	1.8
*Total fluid balance (intraoperative; day 1; 2), ml*				
Mean (SD)	12,920 (6411)	2291 (5449)	<0.001	5.4
*Blood products substitution, n (%)*	17 (65.4)	9 (81.8)	0.265	33.9
*Intraoperative VIS-score*				
Mean (SD)	17.7 (16.5)	8.7 (16.0)	0.044	0.0
*Total VIS-score (intraoperative, day 1 and 2)*				
Median [IQR]	84.6 [43.7, 108.4]	27.2 [17.2, 56.7]	0.004	0.0
*ECMO perioperative, n (%)*	6 (21.4)	1 (3.6)	0.1	
*Duration of intubation, days*				
Mean (SD)	7.1 (7.2)	4.2 (6.2)	0.1	5.4
Median [IQR]	7 [2,8]	2 [1,4]	0.01	5.4
*ICU LOS, days*				
Mean (SD)	14.7 (13.12)	8.9 (9.5)	0.06	0.0
Median [IQR]	10 [5, 19.25]	4.5 [4, 10.2]	0.02	
*Hospital LOS, days*				
Mean (SD)	27.1 (15.1)	18.0 (9.3)	0.008	0.0
Median [IQR]	24.5 [16,32]	14 [12, 19.5]	0.002	0.0
*SIRS, n (%)*	13 (46.4)	5 (17.9)	0.044	0.0
*In-hospital mortality, n (%)*	2 (7.1)	1 (3.6)	1.000	0.0
*30-day mortality, n (%)*	2 (7.1)	1 (3.6)	1.000	0.0
*90-day mortality, n (%)*	2 (7.1.)	1 (3.8)	1.000	0.0
*Morbidity, n (%)*	24 (85.7)	22 (78.6)	0.729	0.0
Minor complications (Grade I-II)***, n (%)	9 (37.5)	12 (54.5)	0.257	0.0
*Major complications (Grade III-IV), n (%)*	15 (62.5)	10 (45.4)	0.375	0.0
Reoperation during hospital stay****	13 (46.4)	7 (28)	0.162	
*NYHA functional class at 6 months, n (%)*				
I	7 (29.2)	14 (56)	0.17	12.5
II	9 (37.5)	7 (28)		
III	6 (25)	4 (16)		
IV	2 (8.3)	0 (0)		

Abbreviations: CPB, cardiopulmonary bypass; ECMO, extracorporeal membrane oxygenation; HA, human albumin; ICU, intensive care unit; IQR, interquartile range; LOS, length of stay; NYHA, New York Heart Association; SD, standard deviation; SIRS systemic inflammatory response syndrome.

* Missing shows the percentage of missing values for that variable.

** Including coronary arterial bypass graft or ascending aortic procedures.

*** Complication grade I-V, according to Clavien-Dindo classification.

**** Reoperations due to complication.

### Primary outcome measure

**Figure 2:**
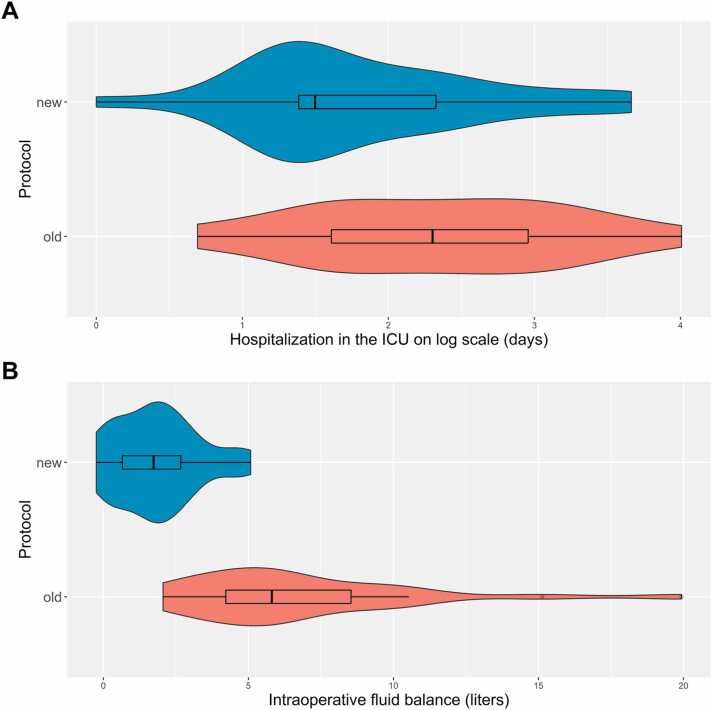
*Descriptive violin and box plots for protocol type—old (green) and new (red)—versus hospitalization in the ICU on log scale **(A)** or intraoperative fluid balance **(B)***. With weak evidence a shorter hospitalization in the ICU was observed with the new protocol (8.9 (9.5) vs 14.7 (13.12) days, *p* = 0.062). ICU, intensive care unit **(A)**. A lower intraoperative fluid balance was observed with the new protocol (mean (standard deviation) 1.854 (1.418) vs 6.764 (4.044) liters, *p* < 0.001) **(B)**.

In the group treated with the new protocol, the median duration of hospitalization in the ICU was shorter compared with the previous protocol (4.5 vs 10 days, Figure 2A), as was the median LOS (14 vs 24.5 days).

### Operative time and CPB-characteristics

Patients treated with the new protocol experienced several differences: operative time (mean 407 vs 451 min), CPB-duration (mean 282 vs 309 min), and a duration of aortic cross-clamping (mean 113 vs 139 min). There was no difference in the duration of circulatory arrest (mean of 48 vs 47 min) or Jamieson classification (Table 3).

### Fluid balance

Intraoperative fluid balance and CPB-balance, as well as total fluid balance of the first 2 days, were all ≥5 times lower with the new protocol (1.85 vs 6.76 liters, −0.39 vs 2.48 liters and 2.29 vs 12.92 liters) (Table 3, Figure 2B). Intraoperative and postoperative variables and outcomes per fluid balance category are presented in Table S3.

### Mortality and morbidity

The postoperative mortality rate at 30 days (Table 3) was 3 out of 56 patients (5%). Among them, two patients (7%) from group A and one patient (4%) from group B died after surgery. This trend remained consistent at 90 days, with no significant difference observed between the two groups.

Causes of in-hospital mortality (*n* = 3) were cardiogenic and septic shock, retroperitoneal and gastrointestinal hemorrhage, and right heart failure following PEA in a patient with AKI.

Morbidities were reported in 24 (86%) patients in group A and in 22 (79%) patients in group B (Table 3). Among patients operated with the new protocol, 12 out of 22 (52%) complications were minor (grade I-II), according to Clavien-Dindo classification, whereas in patients with the old protocol, 9 out of 24 (37%) complications were minor and 15 out of 24 (62%) were major. There was no evidence for a difference in overall morbidity or in minor and major complications. However, systemic inflammatory response syndrome (SIRS) was the most frequent single complication in group A, indicating a distinction between the groups with 13 cases (46%) vs five cases (18%). Atrial fibrillation and reoperation rates were equal between the two groups (Table S4).

## Multivariable regression analysis

### Influence of protocol type and operative time on CPB-intraoperative fluid balance

With the implementation of the new protocol and holding operative time constant, there is an average decrease in intraoperative fluid balance by 3.7 liters [95% CI: −5.2, −2.1], *p* < 0.0001, compared with the old protocol (Table 4, Figure 4A).

**Table 4:** Multivariable Regression Analysis of the Influence of Protocol Change, Operative Time, and Intraoperative Fluid Balance on Short-Intermediate- and Long-Term Outcomes of PEA Surgery

A. Influence of protocol type and operative time on intraoperative fluid balance				

Outcome: Intraoperative fluid balance*	*n* = 53	Coefficient	[95% CI]	*p*-value
	New protocol	−3.68	[−5.22, −2.14]	<0.0001
	Operative time (hours)	1.4	[0.80, 2.00]	<0.0001

B. Influence of intraoperative fluid balance on outcomes of interest				

Outcome: ICU LOS	*n* = 53	Coef, log scale	[95% CI]	*p*-value
	Fluid balance (liters)	0.10	[0.04, 0.16]	0.0011
Outcome: Duration of intubation	*n* = 50	Coef, log scale	[95% CI]	*p*-value
	Fluid balance (liters)	0.13	[0.05, 0.21]	0.0013
Outcome: Hospital LOS	*n* = 53	Coef, log scale	[95% CI]	*p*-value
	Fluid balance (liters)	0.05	[0.01, 0.09]	0.008
Outcome: VIS-score	*n* = 53	Coef, log scale	[95% CI]	*p*-value
	Fluid balance (liters)	0.11	[0.06, 0.17]	<0.0001
Outcome: Morbidity	*n* = 53	OR	[95% CI]	*p*-value
	Fluid balance (liters)	1.61	[1.05, 3.29]	0.09
Outcome: NYHA I/II versus III/IV after 6 months	*n* = 47	OR	[95% CI]	*p*-value
	Fluid balance (liters)	0.97	[0.71, 1.29]	0.82A

C. Influence of protocol type and operative time on outcomes of interest				

Outcome: ICU LOS	*n* = 55	Coef, log scale	[95% CI]	*p*-value
	New protocol	−0.29	[−0.74, 0.16]	0.20
	Operative time (hours)	0.33	[0.16, 0.50]	0.0003
Outcome: Duration of intubation	*n* = 55	Coef, log scale	[95% CI]	*p*-value
	New protocol	−0.42	[−0.92, 0.07]	0.09
	Operative time (hours)	0.33	[0.13, 0.53]	0.002
Outcome: Hospital LOS	*n* = 55	Coef, log scale	[95% CI]	*p*-value
	New protocol	−0.32	[−0.58, −0.06]	0.02
	Operative time (hours)	0.13	[0.03, 0.23]	0.01
Outcome: VIS-score	*n* = 55	Coef, log scale	[95% CI]	*p*-value
	New protocol	−0.44	[−0.95, 0.06]	0.08
	Operative time (hours)	0.2	[0.01, 0.39]	0.04
Outcome: Morbidity	*n* = 55	OR	[95% CI]	*p*-value
	New protocol	1.06	[0.15, 7.14]	0.95
	Operative time (hours)	5.84	[1.94, 28.33]	0.0076
Outcome: NYHA I/II versus III/IV after 6 months	*n* = 55	OR	[95% CI]	*p*-value
	New protocol	0.52	[0.11, 2.21]	0.39
	Operative time (hours)	0.93	[0.46, 1.70]	0.82

Abbreviations: CI, confidence interval; Coef, log scale: estimated regression coefficient reported on the logarithmic scale of the outcome variable; ICU, intensive care unit; LOS, length of stay; NYHA, New York Heart Association; OR, odds ratio; VIS, vasoactive-inotropic score.

Both the new protocol and shorter operative time lead to lower intraoperative fluid balance. A. Influence of protocol type and operative time on intraoperative fluid balance. B. Influence of intraoperative fluid balance on outcomes of interest. C. Influence of protocol type and operative time on outcomes of interest.

* Fluid balance, intraoperative fluid balance presented per 1 liter.

### Influence of CPB-intraoperative fluid balance on outcomes of interest

The impact of intraoperative fluid balance alteration on short- and long-term outcomes is analyzed in several regression models as shown in Table 4. When the intraoperative fluid balance increases by 1 liter, several outcomes are affected: duration of hospitalization in the ICU increases by 11% [95% CI: 4%, 18%], *p* = 0.0011, intubation duration increases by 14% [95% CI: 6%, 23%], *p* = 0.0013, LOS increases by approximately 5% [95% CI: 1%, 9%], *p* = 0.008, VIS in days 1 and 2 after surgery increases by approximately 12% [95% CI: 6%, 18%], *p* < 0.0001 and odds of morbidity increase by approximately 61% [95% CI: 5%, 229%], *p* = 0.09. There is no evidence for an association of NYHA-class after 6 months and intraoperative fluid balance.

### Influence of protocol type and operative time on outcomes of interest

**Figure 3:**
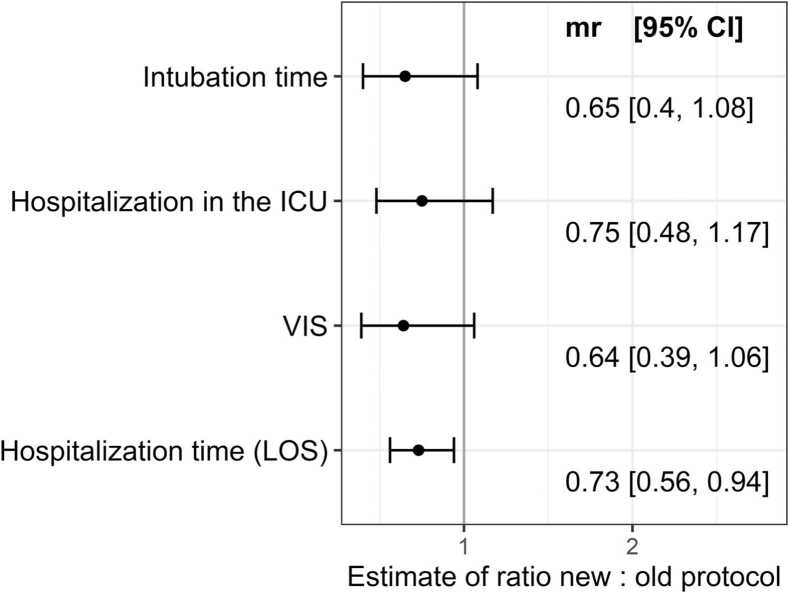
*Forest plot summarizing the effects of protocol change on outcomes as derived from linear regression models with outcomes on logarithmic scale and considering the potential confounders NYHA-class before surgery and operative time.* Mean ratio (MR) and their 95% CI comparing the new with the old protocol are reported. ICU, intensive care unit; VIS, vasoactive-inotropic score; LOS, length of stay; CI, confidence interval; MR, mean ratio; NYHA, New York Heart Association.

Table 4 and Figure 3 show the estimated effect of protocol type on the outcomes by correcting for the potential confounders such as operative time and NYHA-class.

### Impact of operative time

Prolonged operative time in group A was strongly associated with adverse outcomes: duration of hospitalization in the ICU increased by 39%, intubation duration increased by 39%, and LOS increased by 14%, all per 1 hour longer operative time. Additionally, the VIS increased by 22%, and the morbidity OR was 5.8.

### Impact of protocol change with the same operative time

When considering the protocol type together with the operative time, evidence for an isolated effect of the protocol change was limited: duration of hospitalization in the ICU decreased by 25% on average, albeit with a wide CI, intubation duration decreased by approximately 60%, VIS decreased by about 35%, and no definitive impact on morbidity was observed. Anyway, there was moderate evidence suggesting a shorter LOS (−27%; [95% CI: −6%, −44%], *p* = 0.02).

**Figure 4:**
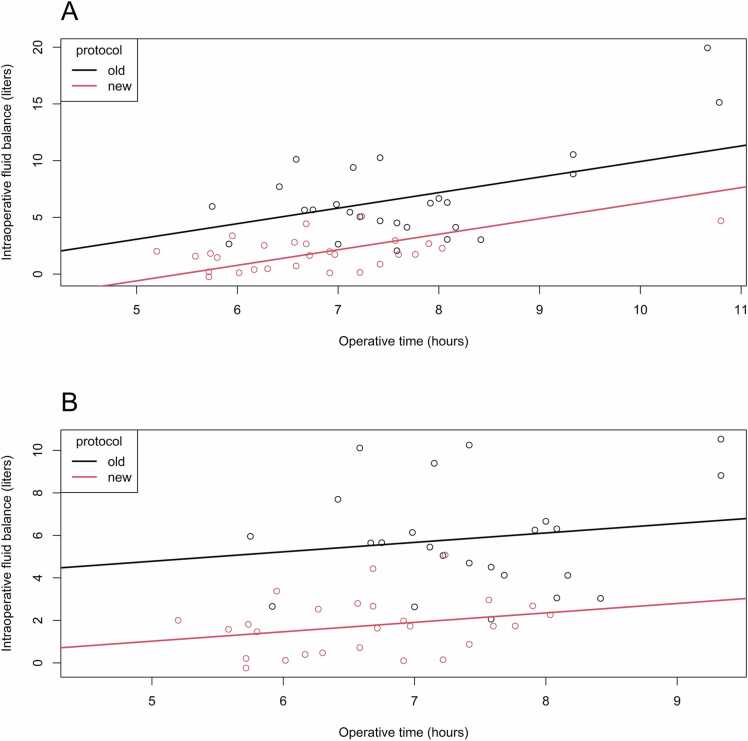
*Relation of intraoperative fluid balance on operative time and protocol type.* Scatter plot with linear regression lines visualizing the increasing intraoperative fluid balance with increasing operative time, separate for patients treated with old protocol (group A) and new protocol (group B). **A**: Plot with all 65 patients included. **B**: Sensitivity analysis: Plot with 53 patients, three patients with a surgery duration of >10 hours are removed.

When performing sensitivity analyses excluding patients with operative times >10 hours, associations between operative time, protocol type, intraoperative fluid balance, and outcomes remained consistent (Figure 4B).

## Discussion

The present study found that implementing a new CPB-priming protocol with 5% HA improved outcomes for patients with CTEPH undergoing PEA. Patients with the new protocol had lower CPB-fluid balance, shorter operative time, shorter duration of hospitalization in the ICU and LOS, and reduced VIS compared with the previous protocol group, when not controlling for operative time.

While ICU LOS is influenced by multiple variables, our statistical models accounted for key confounders such as operative time and preoperative PVR. The observed improvements in outcomes, including reduced ICU LOS and intubation duration, were strongly associated with lower intraoperative fluid balance achieved with the new protocol. Statistical analysis confirmed that fluid balance mediated these benefits, highlighting the critical role of optimized fluid management during CPB.

The protocol change shortened hospitalization time, independent of operative time, by 27%. This suggests that the new protocol helps mitigate fluid shifts during surgery, reducing the need for extensive circulatory support and blood transfusions. Mortality and morbidity rates were similar between groups.

ICU protocols evolved during the study period to prioritize early extubation, contributing to shorter intubation times. Although no significant differences were observed in overall morbidity or complication rates, the protocol change facilitated faster extubation and reduced ICU stays, likely due to improved pulmonary and systemic fluid dynamics.

The data presented suggest that fluid shifts occurring during CTEPH disease are exacerbated during PEA by CPB-induced fluid overload and extravasation. The data further indicate that the new protocol significantly (>5-fold) reduced intraoperative and postoperative CPB-fluid balance, mainly by preserving capillary integrity and ensuring sufficient urine output.

The addition of 5% HA to the CPB priming solution significantly reduced intraoperative fluid balance, possibly by maintaining colloid oncotic pressure, reducing capillary leakage, preventing excessive fluid extravasation, and mitigating systemic inflammation. This effect was observed despite consistent CPB practices over the study period, including the use of hemofiltration, steroids, mannitol, and standardized transfusion triggers. The marked reduction in fluid overload highlights the potential for even small changes in CPB prime composition to influence postoperative outcomes, particularly in high-risk surgical populations such as PEA for CTEPH. These effects were particularly beneficial in the context of PEA, where heightened vascular permeability and DHCA exacerbate fluid management challenges.Significant hemodilution produced by prime solutions in cardiac surgery can lead to electrolyte imbalances, reduced clotting factors and plasma proteins, stress hormone release, and complement activation.^23^, ^24^ This hemodilution decreases colloidal oncotic pressure, favoring fluid shifting into tissues and potentially causing adverse outcomes.^25^ Therefore, minimizing priming volumes and avoiding transfusions is recommended.

The observed 1.3-liter reduction in intraoperative crystalloid administration likely contributed to the overall reduction in intraoperative fluid balance. Although seemingly modest, this change aligns with the colloid's ability to reduce capillary permeability and fluid extravasation, mitigating pulmonary and systemic fluid overload. The cumulative effect of reduced fluid accumulation, rather than crystalloid alone, is likely responsible for the observed differences in postoperative outcomes.

While crystalloid solutions are cost-effective and readily available, they significantly reduce colloidal oncotic pressure.^26^, ^27^ Albumin, a natural colloid, maintains plasma oncotic pressure, prevents capillary leakage, and improves organ function.^28^ However, it is expensive, less accessible, and carries a risk of anaphylaxis.^27^

The advantages of colloids in priming solution versus crystalloids on volume effect during CPB in cardiac surgery have been described.^29^ In a randomized single-center trial, Svendsen et al explored HES as a priming solution during cardiac surgery.^30^ They found that using HES in CPB-priming reduced fluid accumulation and improved early postoperative cardiac function, as evidenced by increased cardiac index.

In general, it is known that perioperative HES can interfere with blood coagulation, causing excessive bleeding and AKI.^31^, ^32^, ^33^, ^34^ Furthermore, two large randomized clinical trials found harmful effects of rapidly degradable HES solutions on renal function for fluid resuscitation in critical intensive care patients.^35^, ^36^

In a recent prospective observational study, Ryhammer et al^37^ used propensity score matching to analyze 17,742 cardiac surgery patients. The authors showed that there was no link between the use of perioperative HES and occurrences of new dialysis, elevation of 30-day and 6-month mortality, or new postoperative ischemic events. However, they acknowledged limitations such as the challenge in distinguishing between colloid and crystalloid administration throughout different phases of patient care, as well as incomplete data on colloid administration.

Since the early 1970s, albumin has been commonly added to extracorporeal circuit prime during cardiac surgery. Its inclusion has been shown to reduce extravascular lung water accumulation by decreasing vascular permeability and pulmonary shunt fraction.^38^, ^39^ HA in the prime can also mitigate the decline in colloid oncotic pressure during bypass, potentially preventing myocardial, pulmonary, intestinal, and cerebral edema.^38^, ^40^ Nevertheless, there is a shortage of extensive randomized controlled trials that directly compare albumin with crystalloid solutions in patients undergoing cardiac surgery. A current randomized clinical trial by Vlasov et al^41^ will demonstrate, in the published study protocol, the effectiveness and safety of HA-solution in patients undergoing cardiac surgery.

The existing literature does not offer a specific formulation preference for priming solution in PEA-procedures.^42^ Over 45% of perfusionists indicate utilizing a combination of crystalloid, colloid, and osmotic components. Additionally, there is no unanimous agreement regarding whether factors such as cerebral protection, safeguarding the pulmonary vascular bed, or maintaining cellular homeostasis impact the choice of prime solution.^42^

In our cohort, the implementation of the new perioperative protocol led to a dramatic decrease in fluid accumulation, which was associated with a lower incidence of postoperative SIRS (5 cases in group B compared with 13 cases in group A). Conversely, for patients following the old protocol, every additional 1,000 ml increase in intraoperative fluid balance was linked to a 60% rise in morbidity.

It is important to acknowledge that the observed differences were influenced by both the protocol change and shorter operative times. Regression analysis confirmed the protocol's independent impact on outcomes, though causality cannot be fully established. Given the unique fluid management needs in PEA compared to general cardiac surgery, the inclusion of albumin in the CPB prime may offer specific advantages, as seen in this cohort. However, further multicenter studies are needed to define its role in broader clinical guidelines.

Interestingly, Delaporte et al,^43^ in recently published propensity-matched analysis involving 202 patients following the presentation of our abstract at ISHLT 2021,^44^ found that using 4% albumin instead of crystalloid for priming of the CPB in patients undergoing PEA did not lower the incidence of pulmonary edema. However, they did observe a significant reduction in acute respiratory distress syndrome within the albumin group (3.5% vs 13.7%, *p* = 0.008).

Stanzel et al,^42^ discovered a considerable variation in prime solutions across different centers, with nearly half using combinations of crystalloid, colloid, and osmotic compounds. In follow-up surveys, some centers shifted towards colloids rather than osmotic agents.

Our results emphasize the benefits of using a priming protocol with 5% HA during CPB, encouraging the need for standardization in practice guidelines. However, there are currently no randomized controlled trials providing a comprehensive examination of perfusion management during PEA for patients with CTEPH. This would be ethically unjustifiable for PEA-classified patients with CTEPH.

The approach taken here entailed incorporating 5%HA as a colloid solution into the CPB-prime and maintenance protocol. This adjustment effectively prevented capillary leak during the procedure and facilitated successful postoperative ventilator weaning. Postoperative mortality rates for PEA can vary based on factors such as patient health, condition severity, surgical team expertise, and complications. Mortality rates have decreased from 20%^45^ to 2.5% in experienced centers^46^ due to improved techniques and care.

Due to its complexity and potential complications, PEA surgery imposes a steep learning curve.^47^ Surgeons benefit from structured training and mentorship, and hospitals should cultivate a culture of continuous learning and quality improvement in PEA-services. Sihag et al indicate that around 20 cases are needed for proficiency, while nearly 100 cases are required for favorable outcomes.^45^ Additionally, patient selection, careful preoperative assessment, and multidisciplinary teamwork are critical factors in optimizing outcomes during the learning curve phase.

The increase in PEA case volume during the study period reflects the center’s evolution from a low- to a moderate-volume center. This raised the potential for confounding from the learning curve and other temporal changes, such as updates to surgical and perfusion materials, logistical workflows, and ERAS principles. However, most of the analyzed procedures were done within the 6 years when the center was a moderate-volume center. In addition, several measures were employed to minimize temporal effects. Multivariable regression analyses adjusted for operative time as a surrogate for surgical complexity and learning curve effects. Sensitivity analyses excluding prolonged surgeries confirmed the independent impact of the protocol change on intraoperative fluid balance and patient outcomes, supporting the robustness of these findings.

In the presented cohort of patients, we would like to emphasize the independence of the results from the learning curve and that all patients were managed by the same experienced team of thoracic surgeons (IO, II), cardiac anaesthesiologists (DB, TH), perfusionists (OS, NN), intensive care physicians (RS), and pneumologists (SU).

The strength of this study is the analysis of the innovative choice of prime and maintenance solution for CPB during PEA in CTEPH patients. Large multicenter trials comparing the benefit of HA versus other fluids,^46^ linking specific perfusion strategies to patient outcomes, are needed to define the best approach for PEA.

## Study Limitations

Using a historical control group cannot eliminate the risk of confounding by unobserved time-dependent factors and evolving practices, including increased surgical experience, changes in materials, and the adoption of ERAS principles, suggesting association rather than causation.^45^ While adjustments for operative time and sensitivity analyses helped mitigate the impact of a learning curve. The impact of a learning curve in the present two cohorts cannot be fully excluded; it becomes obvious that currently (in 2024) the mortality rate achieved is 2.34%, in comparison with the 5% of the whole cohort described in the study. Propensity-score matching may exclude special cases, although in this study the inclusion rates for both protocols were relatively high, minimizing this concern. Additionally, due to the small number of eligible patients in each cohort, a power calculation was not conducted.

## Conclusions

The addition of 5% HA to the CPB priming solution significantly reduced intraoperative fluid balance and improved fluid management during PEA surgery for CTEPH. These changes contributed to faster postoperative recovery, including shorter intubation duration and ICU stays and lower catecholamine requirements. While shorter operative times also improved outcomes, the protocol change’s benefits were primarily mediated through enhanced fluid dynamics rather than a direct effect on surgical efficiency. Furthermore, the substantial reduction in total fluid balance under the new protocol minimized complications associated with fluid overload, such as pulmonary and systemic inflammation, despite the absence of significant differences in major morbidity rates. These findings support the consideration of colloid solutions in CPB protocols for PEA, though further studies are needed to validate their use in broader cardiac surgery populations.

## Financial support

Isabelle Opitz: Roche (Institutional Grant and Speakers Bureau), Roche Genentech (Steering Committee), AstraZeneca (Advisory Board and Speakers Bureau), MSD (Advisory Board), BMS (Advisory Board), Medtronic (Institutional Grant and Advisory Board), Intuitive (Proctorship). Silvia Ulrich: Grants from the Swiss National Science Foundation, Zurich and Swiss Lung League, unrestricted grants and personal fees from Janssen SA, MSD SA, Orpha Swiss, Gebro and Novartis, all unrelated to the present work.

## Author contributions

K.F. participated in writing of the manuscript and critical care revision of the article, analysis and interpretation of results, performance of literature search and final approval of the manuscript. B.D., T.H., I.I., and S.U. participated in critical review of the manuscript. NNG participated in data collection and critical review of the manuscript. M.H-T. and B.B. participated in the data collection and critical review of the manuscript. M.H. participated in statistical data analysis, interpretation of results and writing of the manuscript. R.S. participated in data collection and critical review of the manuscript. I.O. participated in conception and research design, analysis and interpretation of results, writing of the manuscript and critical review of the manuscript, performance of the research and final approval of the manuscript. All authors discussed the results and contributed to the final manuscript.

## Declaration of Generative AI and AI-Assisted Technologies in the Writing Process

AI-assisted tools has been used to improve the readability and language of the work.

## Declaration of Competing Interest

The authors declare the following financial interests/personal relationships which may be considered as potential competing interests: Isabelle Opitz reports a relationship with Roche that includes: funding grants. Isabelle Opitz reports a relationship with AstraZeneca that includes: consulting or advisory. Isabelle Opitz reports a relationship with MSD that includes: consulting or advisory. Isabelle Opitz reports a relationship with BMS that includes: consulting or advisory. Isabelle Opitz reports a relationship with Medtronic that includes: consulting or advisory and funding grants. Isabelle Opitz reports a relationship with Intuitive that includes: consulting or advisory and speaking and lecture fees. Isabelle Opitz reports a relationship with Sanofi that includes: speaking and lecture fees. Isabelle Opitz reports a relationship with Regeneron that includes: consulting or advisory. Isabelle Opitz reports a relationship with XVIVO that includes: funding grants. Isabelle Opitz reports a relationship with Siemens that includes: speaking and lecture fees. Isabelle Opitz reports a relationship with Astellas that includes: speaking and lecture fees. Silvia Ulrich reports a relationship with Janssen SA that includes: speaking and lecture fees. Silvia Ulrich reports a relationship with MSD that includes: speaking and lecture fees. Silvia Ulrich reports a relationship with Orpha Swiss that includes: speaking and lecture fees. Silvia Ulrich reports a relationship with Gebro that includes: speaking and lecture fees. Silvia Ulrich reports a relationship with Novartis that includes: speaking and lecture fees. If there are other authors, they declare that they have no known competing financial interests or personal relationships that could have appeared to influence the work reported in this paper.

## Footnotes

Appendix A Supplementary data associated with this article can be found in the online version at doi:10.1016/j.jhlto.2025.100253.
