# Supplementary material for: Fluid management of cardiopulmonary bypass during pulmonary endarterectomy for CTEPH patients impacts perioperative outcome
Source: JHLT Open. 2025 Jun 4;9:100253. doi: 10.1016/j.jhlto.2025.100253 (PMC12268579; doi:10.1016/j.jhlto.2025.100253)
Supplement: Supplementary file 1 — Supplementary material [file mmc1.docx]

**Fluid Management of Cardiopulmonary Bypass During Pulmonary Endarterectomy for Patients with CTEPH Impacts Perioperative Outcome**

**Supplemental Material**

**Table S1. Comparative preoperative characteristics of the matching variables in full, unmatched cohort.**

|  | **Old protocol**  **n=45** | **New protocol**  **n=28** | **p-value** | **SMD*** |
| --- | --- | --- | --- | --- |
| **Male sex, n (%)** | 25 (55.6) | 18 (64.3) | 0.622 | 0.179 |
| **Age, years**  Mean (SD) | 58.73 (15.90) | 58.93 (14.03) | 0.958 | 0.013 |
| **PVR**, WU**  Mean (SD) | 6.52 (2.90) | 6.69 (3.03) | 0.816 | 0.056 |

* standardized mean difference (SMD)

**PVR, pulmonary vascular resistance expressed in Wood units = mmHg/L/min**Table S2. Comparative, detailed presentation of intra-and postoperative catecholamine administration in matched dataset according to old and new protocol.**

| **Catecholamine administration (µg/min)** | **Old protocol**  **Group A**  **n=28** | **New protocol**  **Group B**  **n=28** | **p-value** |
| --- | --- | --- | --- |
| **Intraoperative Noradrenaline requirement**  Mean (SD) | 12.12 (9.66) | 6.46 (10.87) | 0.044 |
| **Intraoperative Adrenaline requirement**  Mean (SD), | 0.68 (1.52) | 0.00 (0.00) | 0.022 |
| **Intraoperative Corotrop requirement**  Mean (SD) | 5.29 (12.80) | 0.18 (0.94) | 0.04 |
| **Intraoperative Dobutamine requirement**  Mean (SD) | 0.00 (0.00) | 3.57 (18.90) | 0.322 |
| **Noradrenaline requirement on day 1.**  Mean (SD) | 23.89 (14.83) | 15.46 (13.82) | 0.032 |
| **Adrenaline requirement on day 1.**  Mean (SD) | 4.21 (18.83) | 0.00 (0.00) | 0.241 |
| **Corotrop requirement on day 1.**  Mean (SD) | 3.32 (5.93) | 0.18 (0.94) | 0.008 |
| **Noradrenaline requirement on day 2.**  Mean (SD) | 25.79 (19.14) | 15.50 (14.30) | 0.027 |
| **Adrenaline requirement on day 2.**  Mean (SD) | 0.89 (3.80) | 0.00 (0.00) | 0.220 |
| **Corotrop requirement on day 2.**  Mean (SD) | 3.82 (6.30) | 0.21 (1.13) | 0.004 |
| **Dobutamine requirement on day 2.**  Mean (SD) | 0.00 (0.00) | 7.14 (37.80) | 0.322 |

*Missing shows the percentage of missing values for that variable

**Table S3. Descriptive presentation of intraoperative and postoperative variables and outcomes per fluid balance category.**

| **Intraoperative fluid balance** | | | | | | |
| --- | --- | --- | --- | --- | --- | --- |
|  | **<2.001 liters** | **2.001-5.0 liters** | **5.001-10.0 liters** | **>10.0 liters** | **p-value** | **Missing*** |
| n= | 17 | 19 | 13 | 5 |  |  |
| **NYHA functional class, n (%)** |  |  |  |  |  |  |
| II | 7 ( 41.2) | 5 (26.3) | 3 ( 25.0) | 2 ( 40.0) | 0.510 | 1.8 |
| III | 7 ( 41.2) | 12 (63.2) | 9 ( 75.0) | 3 ( 60.0) |  |  |
| IV | 3 ( 17.6) | 2 (10.5) | 0 ( 0.0) | 0 ( 0.0) |  |  |
| **sPAP, mmHg**  Mean (SD) | 66.82 (18.61) | 68.74 (14.34) | 69.31 (12.68) | 62.60 (17.57) | 0.850 | 0.0 |
| **Weight**, kg**  mean (SD), | 89.77 (30.97) | 84.17 (20.38) | 81.65 (18.72) | 73.64 (20.22) | 0.569 | 0.0 |
| **Operative time, hours**  Mean (SD) | 6.54 (0.72) | 7.34 (1.22) | 7.28 (0.90) | 8.96 (1.90) | 0.001 | 0.0 |
| **Intraoperative fluid balance, liters**  Mean (SD) | 0.96665 (0.76566) | 3.22547 (0.91056) | 6.46938 (1.366.03) | 13.1928 (4.313.11) | <0.001 | 3.6 |
| **Total fluid balance (intraoperative; day 1. 2.), liters**  Mean (SD) | 0.64306 (4.07111) | 6.71274 (6.52948) | 12.70367 (4.70586) | 19.24540 (6.71197) | <0.001 | 5.4 |
| **Intraoperative VIS**  Mean (SD) | 7.05 (13.83) | 7.51 (7.49) | 26.12 (20.72) | 24.82 (21.65) | 0.001 | 0.0 |
| **Total VIS-score (intraoperative, day 1, 2.)**  Mean (SD) | 42.10 (55.47) | 58.68 (37.69) | 97.10 (49.33) | 159.20 (127.09) | 0.001 | 0.0 |
| **Duration of intubation, days**  Mean (SD), | 4.41 (7.23) | 4.67 (3.50) | 7.25 (8.90) | 13.50 (6.45) | 0.076 | 5.4 |
| **Duration of hospitalization in the ICU, days**  Mean (SD) | 6.35 (4.55) | 11.53 (10.04) | 17.15 (17.88) | 20.20 (6.94) | 0.027 | 0.0 |
| **Hospitalization time (LOS ), days**  Mean (SD) | 16.29 (6.71) | 21.79 (9.77) | 30.77 (20.79) | 26.60 (8.59) | 0.024 | 0.0 |
| **In-hospital mortality, n (%)** | 0 ( 0.0) | 1 ( 5.3) | 0 ( 0.0) | 2 ( 40.0) |  |  |
| **30-day mortality, n (%)** | 0 ( 0.0) | 1 ( 5.3) | 0 ( 0.0) | 2 ( 40.0) |  |  |
| **90-day mortality, n (%)** | 0 ( 0.0) | 1 ( 5.3) | 0 ( 0.0) | 2 ( 40.0) |  |  |
| **Morbidity, n (%)** | 14 ( 82.4) | 16 (84.2) | 13 (100.0) | 5 (100.0) |  |  |
| **NYHA functional class at 6 months postoperative, n (%)** |  |  |  |  |  |  |
| I | 9 ( 60.0) | 7 (38.9) | 4 ( 36.4) | 1 ( 33.3) | 0.780 | 12.5 |
| II | 4 ( 26.7) | 5 (27.8) | 4 ( 36.4) | 2 ( 66.7) |  |  |
| III | 2 ( 13.3) | 5 (27.8) | 2 ( 18.2) | 0 ( 0.0) |  |  |
| IV | 0 ( 0.0) | 1 ( 5.6) | 1 ( 9.1) | 0 ( 0.0) |  |  |

*Missing shows the percentage of missing values for that variable

**Before surgery

**Table S4. Postoperative complications in matched dataset according to old and new protocol.**

**Multiple complications per patient were possible.**

| **Postoperative complications** | **Old protocol**  **Group A**  **n=28** | **New protocol**  **Group B**  **n=28** | **p-value** |
| --- | --- | --- | --- |
| **Cardiac** |  |  |  |
| Persistent pulmonary hypertension, (%) | 2 (7.4) | 3 (10.7) | 1.000 |
| Rethrombosis, (%) | 0 (0.0) | 1 (3.7) | 0.985 |
| Right ventricular failure, (%) | 5 (17.9) | 2 (7.1) | 0.419 |
| Atrial fibrillation, (%) | 12 (42.9) | 10 (35.7) | 0.784 |
| Supraventricular tachycardia, (%) | 2 (7.1) | 1 (3.6) | 1.000 |
| Cardiac arrest, (%) | 0 (0.0) | 1 (3.6) | 1.000 |
| Pericardial effusion, (%) | 2 (7.1) | 3 (10.7) | 1.000 |
| Cardiac tamponade, (%) | 2 (7.1) | 1 (3.6) | 1.000 |
| **Pulmonary** |  |  |  |
| Lung reperfusion edema, (%) | 6 (22.2) | 3 (10.7) | 0.430 |
| Reintubation, (%) | 5 (17.9) | 1 (3.6) | 0.195 |
| Pneumonia, (%) | 9 (32.1) | 6 (21.4) | 0.546 |
| Intrapulmonary bleeding, (%) | 2 (7.4) | 0 (0.0) | 0.455 |
| Acute Respiratory Distress Syndrome (ARDS), (%) | 0 (0.0) | 1 (3.6) | 1.000 |
| Tracheostomy, (%) | 6 (21.4) | 2 (7.1) | 0.252 |
| Respiratory failure, (%) | 3 (10.7) | 1 (3.6) | 0.604 |
| Hematothorax, (%) | 6 (21.4) | 2 (7.1) | 0.252 |
| Pneumothorax, (%) | 4 (14.3) | 1 (3.6) | 0.349 |
| Pulmonary embolism, (%) | 1 (3.6) | 1 (3.6) | 1.000 |
| **Neurologic** |  |  |  |
| Encephalopathy, (%) | 2 (7.1) | 3 (10.7) | 1.000 |
| Subdural hematoma, (%) | 1 (3.6) | 0 (0.0) | 1.000 |
| Delir, (%) | 10 (35.7) | 8 ( 28.6) | 0.775 |
| Stroke, n (%) | 4 (14.3) | 6 (21.4) | 1.000 |
| Critical illness polyneuropathy, (%) | 3 (10.7) | 2 (7.1) | 1.000 |
| **Infectious/Inflammatory** |  |  |  |
| Sepsis, (%) | 6 (21.4) | 2 (7.1) | 0.252 |
| Wound infection, (%) | 2 ( 7.1) | 0 ( 0.0) | 0.471 |
| Systemic Inflammatory Response Syndrome (SIRS), (%) | 13 (46.4) | 5 (17.9) | 0.022 |
| **Technical** |  |  |  |
| Arteriotomy rupture, (%) | 1 (3.6) | 0 (0.0) | 1.000 |
| Instability of the sternum, (%) | 0 (0.0) | 1 (3.6) | 1.000 |
| **Others** |  |  |  |
| Acute renal failure, (%) | 9 (32.1) | 7 (25.0) | 0.767 |
| Liver dysfunction, (%) | 1 (3.6) | 1 (3.6) | 1.000 |
| Ischemic bowel, (%) | 1 (3.6) | 0 (0.0) | 1.000 |
| Coagulopathy, (%) | 1 ( 3.6) | 0 (0.0) | 1.000 |
| Thrombopenia, (%) | 4 ( 14.3) | 1 (3.6) | 0.349 |
