# Supplementary material for: Fluid management of cardiopulmonary bypass during pulmonary endarterectomy for CTEPH patients impacts perioperative outcome
Source: JHLT Open. 2025 Jun 4;9:100253. doi: 10.1016/j.jhlto.2025.100253 (PMC12268579; doi:10.1016/j.jhlto.2025.100253)
Supplement: Supplementary file 2 — Supplementary material [file mmc2.docx]

**Title of the manuscript**

**Impact of Fluid Management During Cardiopulmonary Bypass on Perioperative Outcomes in CTEPH Patients Undergoing Pulmonary Endarterectomy: A Retrospective Cohort Study**

**Running Title**

**The Choice of Priming Solution for Cardiopulmonary Bypass during Pulmonary Endarterectomy for CTEPH Patients Has Important Impact***

**Authors:** Furrer K^1^, MD, Bettex D^2^, MD, Horisberger T^2^, Inci I^1^, MD, Nagaraj NG^3^, Morselli H-T^1^, Battilana B^1^, Schuepbach R^4^, MD, Ulrich S^5^, MD, Hebeisen M^1,6^, MSc, Opitz I^1^, MD

**Author Affilations:**

^1^Department of Thoracic Surgery, University Hospital Zurich, University of Zurich, Zurich, Switzerland

^2^Institute of Anesthesiology, University Hospital Zurich, Zurich, Switzerland

^3^Department of Perfusion, University Hospital Zurich, Zurich, Switzerland

^4^Institute of Intensive Care Medicine, University and University Hospital Zurich

^5^Department of Pulmonology, University Hospital Zurich, Zurich, Switzerland

^6^ Department of Biostatistics, Epidemiology, Biostatistics and Prevention Institute, University of Zurich, Zurich, Switzerland

**Corresponding Author:**

Isabelle Opitz, MD FEBTS;

Department of Thoracic Surgery; University Hospital Zurich; University of Zurich;

Raemistrasse 100; 8091 Zurich; Switzerland

**Email:** [isabelle.schmitt-opitz@usz.ch](file:///\\\\fs-home\\fuka$\\Desktop\\CTEPH\\isabelle.schmitt-opitz@usz.ch)

Phone: +41 44 255 88 04

*Presented during 41st Annual Congresses of International Society for Heart and Lung Transplantation (ISHLT) 2021

**Supplementary Material**

**STROBE Checklist for Observational Cohort Study**

Title and Abstract

- Study Design: The study design (e.g., cohort, case-control, cross-sectional) is explicitly stated in the title or abstract.
- Informative Abstract: Abstract summarizes objectives, design, setting, participants, methods, main results (including effect sizes and confidence intervals), and conclusions

Introduction

- Background/Rationale: Scientific background and rationale for the investigation clearly described.
- Objectives: Specific objectives and hypotheses stated.

Methods

- Study Design: Key elements of study design (e.g., prospective vs. retrospective) stated early in the paper.
- Setting: Description of setting, locations, and relevant dates (e.g., recruitment, exposure, follow-up).
- Participants:
  - Eligibility criteria stated.
  - Methods of participant selection described.
  - Follow-up procedures outlined, if applicable.
- Variables: All outcomes, exposures, predictors, confounders, and effect modifiers clearly defined.
- Data Sources/Measurement:
  - Sources of data (e.g., medical records, patient questionnaires).
  - Assessment methods and tools described and justified.
- Bias: Efforts to address potential sources of bias (e.g., selection bias, measurement bias) described.
- Study Size: How study size was determined explained (e.g., power calculation, available data).
- Statistical Methods:
  - Methods for controlling confounding (e.g., multivariable models).
  - Approaches for dealing with missing data.
  - Methods for examining subgroups and interactions.
  - Sensitivity analyses, if conducted, described.

Results

- Participants:
  - Flow of participants through the study (e.g., via flowchart).
  - Number of participants at each stage (eligibility, included, followed up, analyzed).
- Descriptive Data:
  - Characteristics of study participants (e.g., demographics, clinical data).
  - Information on exposures and confounders.
- Outcome Data: Report numbers of outcome events or summary measures over time.
- Main Results:
  - Unadjusted and adjusted estimates with precision (e.g., 95% CI).
  - Clarification of which confounders were adjusted for.
- Other Analyses: Report results of subgroup analyses, interactions, and sensitivity analyses.
- Deviations: Explanation of any changes or deviations from the original analysis plan.

Discussion

- Key Results: Summary of main findings with respect to the study objectives.
- Limitations: Discussion of limitations, including potential sources of bias or imprecision.
- Interpretation: Interpretation consistent with results and contextualized within existing literature.
- Generalisability: Assessment of external validity and relevance to other populations/settings.

Additional Information

- Ethical Approval: Statement of ethics approval and consent procedures.
- Funding: Sources of funding and role of funders disclosed.
- Conflicts of Interest: Declaration of any potential conflicts of interest.
- Authors' Contributions: Individual author roles described (e.g., conceptualization, data analysis, writing).

Refrerence

von Elm E, Altman DG, Egger M, Pocock SJ, Gøtzsche PC, Vandenbroucke JP: The Strengthening the Reporting of Observational Studies in Epidemiology (STROBE) statement: guidelines for reporting observational studies. Lancet 2007;370:1453-7.
